# α-Conotoxin ImI-modified polymeric micelles as potential nanocarriers for targeted docetaxel delivery to α7-nAChR overexpressed non-small cell lung cancer

**DOI:** 10.1080/10717544.2018.1436097

**Published:** 2018-02-09

**Authors:** Dong Mei, Libo Zhao, Binlong Chen, Xiaoyan Zhang, Xiaoling Wang, Zhiying Yu, Xin Ni, Qiang Zhang

**Affiliations:** a Beijing Children’s Hospital, Capital Medical University, National Center for Children’s Health, Beijing, PR China;; b State Key Laboratory of Natural and Biomimetic Drugs, School of Pharmaceutical Sciences, Peking University, Beijing, PR China;; c Department of Pharmacy, Peking University People’s Hospital, Beijing, PR China

**Keywords:** α-Conotoxin ImI, α7-nAChR, non-small cell lung cancer (NSCLC), PEG-DSPE micelles, targeted delivery

## Abstract

A micelle system modified with α-Conotoxin ImI (ImI), a potently antagonist for alpha7 nicotinic acetylcholine receptor (α7-nAChR) previously utilized for targeting breast cancer, was constructed. Its targeting efficiency and cytotoxicity against non-small cell lung cancer (NSCLC) highly expressing α7-nAChR was investigated. A549, a non-small cell lung cancer cell line, was selected as the cell model. The cellular uptake study showed that the optimal modification ratio of ImI on micelle surface was 5% and ImI-modification increased intracellular delivery efficiency to A549 cells via receptor-mediated endocytosis. Intracellular Ca^2+^ transient assay demonstrated that ImI modification led to enhanced molecular interaction between nanocarriers and A549 cells. The *in vivo* near-infrared fluorescence imaging further revealed that ImI-modified micelles could facilitate the drug accumulation in tumor sites compared with non-modified micelles via α7-nAChR mediation. Moreover, docetaxel (DTX) was loaded in ImI-modified nanomedicines to evaluate its *in vitro* cytotoxicity. As a result, DTX-loaded ImI-PMs exhibited greater anti-proliferation effect on A549 cells compared with non-modified micelles. Generally, our study proved that ImI-modified micelles had targeting ability to NSCLC in addition to breast cancer and it may provide a promising strategy to deliver drugs to NSCLC overexpressing α7-nAChR.

## Introduction

Lung cancer, the most frequently diagnosed cancer, is the leading cause of cancer-related mortality among males and also the second leading cause of cancer death among females worldwide (Torre et al., 2015, 2016). There are two main types of lung cancer: non-small cell lung cancer (NSCLC) and small cell lung cancer. As reported by national cancer institute (NIH), in 2017, an estimated 222,500 new cases of lung cancer will be diagnosed, of which the majority of patients are NSCLC, with an overall 5-year survival of 18.1% for all stages (Surveillance Epidemiology and End Results Program, 2014). NSCLC shows a great molecular heterogeneity in which several pathways are believed to simultaneously and actively lead to tumorigenesis. Therefore, novel strategies that target-specific pathways associated with apoptosis, cell proliferation, angiogenesis, and other mechanisms have been proposed as unique therapeutic selections for NSCLC (Besse et al., 2007; Sato et al., 2007).

Previous studies positively suggested that the nicotinic acetylcholine receptors (nAChRs) play a significant role in NSCLC predisposition and natural history (Hung et al., 2008; Thorgeirsson et al., 2008). It has been largely demonstrated that among different subtypes of nAChRs, the homomeric pentamer α7-nAChR, composed of five α7-subunits symmetrically arranged around a central ion pore, are responsible for cell growth and tumor progression as well as cell death in NSCLC (Cesario et al., 2012; Zhang et al., 2016). It mediates the proliferative, pro-angiogenic, and pro-metastatic activities of nicotine in human NSCLC (Singh et al., 2011; Schuller, 2012). α7-nAChR causes direct activation of signaling kinases and phosphatases, such as the Rb–Raf-1/phospho-ERK/phospho-p90RSK pathway, thus promoting the progression of human lung cancers. Data from several laboratories have shown that the α7-nAChR antagonists could be considered as potential anticancer agents (Trombino et al., 2004; Russo et al., 2006; Grozio et al., 2008; Paleari et al., 2008a,b), since antagonists (e.g. d-tubocurarine or snake’s long neurotoxin) have an inhibitory effect on tumor growth by binding to α7-nAChR (Cesario et al., 2004; Grozio et al., 2007; Catassi et al., 2008; Grozio et al., 2008; Paleari et al., 2008a,b).

However, there are very few research papers that studied the effect of α7-nAChR used as a molecular target for drug delivery system (DDS) in the treatment of human diseases. In addition, the mechanisms underlying the increased cellular uptake in tumor cells overexpressing α7-nAChR remain to be fully understood. One research work reported that a new targeting molecule of α7-nAChR, a neurotoxin candoxin-derived peptide, could efficiently deliver drug to the intracranial glioblastoma (Zhan et al., 2011). Furthermore, Kuang-Kai Liu revealed that nanodiamond conjugated with alpha-bungarotoxin (α-BTX) provides a visual system by binding of α-BTX to α7-nAChR (Liu et al., 2008). However, all these studies seldom focused on NSCLC, regardless of its wide application potential in the areas of tumor diagnosis, gene delivery, and chemotherapeutic drugs delivery. Whether α7-nAChR is a valuable molecular target of DDS for NSCLC therapy remains an open question, since NSCLC cells also highly express α7-nAChR.

α-Conotoxin ImI (ImI), a conus peptide containing 12 amino acids and 2 disulfide bonds with C-terminal amidated, was originally purified from the venom of *Conus imperialis* (Pereira et al., 1996). ImI is a natural ligand of α7-nAChR with high selectivity, specificity and potency (Ulens et al., 2006; Yu et al., 2011, 2012). In addition to its small size and relative ease of synthesis, structural stability and its ability to specifically target α7-nAChR have made it a valuable molecular probe as well as drug lead (Gehrmann et al., 1999; Kiss et al., 2014). This venom has previously been used in the pharmacological and functional characterization of α7-nAChR, or for inhibiting nicotine action (López et al., 1998; Ellison et al., 2004; Baxter et al., 2014). Compared with currently widely used macromolecular antibodies, the small toxins have a unique advantage for targeting ligands to delivery drugs, since small peptides can overcome the limitations of poor tumor penetration and cellular uptake of antibody when introduced *in vivo* (Aina et al., 2002). Generally, ImI may be a potential tool for tumor targeting therapy and diagnosis because of its specific binding and other natural properties. However, to the best of our knowledge, a direct targeting effect of venom components as targeting ligands to delivery drugs has been rarely investigated and demonstrated. Our group previously proved the excellent targeting capability of α-conotoxin ImI to MCF-7 human breast cancer cells via active binding to α7-nAChR (Mei et al., 2015), while it is unknown whether ImI could be used as a targeting peptide guiding DDS to NSCLC cells.

Based on all of the above, our work aims to investigate the targeting potential of ImI-modified nanomedicines for the treatment of α7-nAChR-overexpressed NSCLC *in vitro* and *in vivo*. First, ImI-modified PEG-DSPE micelles loaded with docetaxel were formulated and characterized, and their targeting potential to human NSCLC A549 cells was evaluated. Both *in vitro* and *in vivo* experiments revealed that the ImI-modified micelles increased the cellular uptake of loaded drugs in A549 cells through α7-nAChR mediation. Real-time intracellular Ca^2+^ transients assay was conducted to further elucidate the mechanisms underlying the increased cellular uptake. Finally, the targeting efficacy of nanocarriers was investigated in A549 cells by assessing cytotoxicity *in vitro.*

## Materials and methods

### Materials

Docetaxel (DTX) was purchased from Bristol-Mayers Squibb (Nutley, NJ). α-Conotoxin ImI (ImI, *M*_w_ = 1352.6) was from China Peptides Co., Ltd (Shanghai, China). Poly (ethylene glycol)-(distearoyl-*sn*-glycero-3-phosphoethanolaminen) (PEG_2000_-DSPE_800_, *M*_w_ = 2922.0) and *N*-hydroxysuccinimidyl-PEG_2000_-DSPE_800_ (NHS-PEG-DSPE, *M*_w_ = 2986.0) were obtained from NOF Corporation (Tokyo, Japan). Coumarin-6 (C6, *λ*_ex_ = 467 nm, *λ*_em_ = 502 nm), sulforhodamine B (SRB), tris base, and trichloroacetic acid (TCA) were all acquired from Sigma-Aldrich (St. Louis, MO). LysoTracker Red (*λ*_ex_ = 577 nm, *λ*_em_ = 590 nm) and Hoechst 33258 (*λ*_ex_ = 352 nm, *λ*_em_ = 460 nm) were products of Molecular Probes Inc. (Eugene, OR). Fluo-3 acetoxy methyl ester (fluo-3/AM, *λ*_ex_ = 488 nm, *λ*_em_ = 526 nm) and fluorescent probe DiR (*λ*_ex_ = 748 nm, *λ*_em_ = 780 nm) were obtained from Biotium, Inc. (Hayward, CA). RPMI-1640 with _L_-glutamine medium, penicillin–streptomycin liquid (100 ×) and 0.25% trypsin were obtained from M&C Gene Technology (Beijing, China). Fetal bovine serum was purchased from GIBCO, Invitrogen Corp. (Carlsbad, CA). Other reagents were of all analytical grade and used as received.

A549 cells were acquired from the Institute of Basic Medical Science, Chinese Academy of Medical Sciences (Beijing, China), and cultured in RPMI1640 medium containing a final concentration of 10% fetal bovine serum and 1% antibiotics (100 U/mL penicillin plus 100 μg/mL streptomycin) at 37 °C under 5% CO_2_ atmosphere.

Female BALB/c mice of 18–20 g in weight were purchased from the Vital River Laboratory Animal Center (Beijing, China). Care and all animal experiments adhered to the guidelines of the Ethics Committee of Peking University.

### Synthesis and characterization of ImI-PEG-DSPE

The targeting material ImI-PEG-DSPE was synthesized according to our previous research (Mei et al., 2015). In brief, NHS-PEG-DSPE and ImI (2:1; molar ratio) were reacting for 120 h in anhydrous *N*,*N'*-dimethylformamide (DMF), adjusted to pH 8.0 with triethylamine. The conjugation efficiency was monitored by thin-layer chromatography (TLC) and reversed-phase high performance liquid chromatography (RP-HPLC, Shimadzu, LC-10AT, Tokyo, Japan). After dialysis and freeze-drying, the targeting copolymer was confirmed by matrix assisted laser desorption/ionization-time of flight (MALDI-TOF) mass spectrometer (Waters, Milford, MA) and ultraviolet and visible (UV–Vis) spectrometer (Beijing puxi, TU-1900, Beijing, China).

### Preparation and characterization of various test nanomedicines

C6 and DiR were used as hydrophobic fluorescent probes to investigate *in vitro* cellular uptake and the *in vivo* distribution of nanomedicines, respectively. DTX was a chemotherapeutic agent to evaluate the cytotoxicity of DTX-loaded micelles on A549 cells. Both blank micelles (PMs and ImI-PMs) and hydrophobic drugs (DTX, C6 or DiR) loaded polymeric micelles (PM-DTX, ImI-PM-DTX, PM-C6, ImI-PM-C6, PM-DiR, and ImI-PM-DiR) were prepared by film hydration method described previously (Mei et al., 2015). The weight ratio of polymers and drugs was 30:1 for DTX, 10,000:1 for C6, and 3000:1 for DiR.

The particle size and the zeta potential of nanomedicines were measured by a dynamic light scattering (DLS) method using Malvern Zetasizer Nano ZS (Malvern, UK). The morphological shape of ImI-PM-DTX was observed by transmission electron microscope (TEM, JEOL, JEM-2100F, Tokyo, Japan).

The encapsulation efficiency (EE) was calculated by the following formula: EE (%) = drug loaded/total drug ×100%. The concentration of C6 or DiR was determined by a fluorescence spectrometer (Cary Eclipse, Varian Corporation, Lake Forest, CA). The EE% of DTX in micelles was quantified by a HPLC system with C18 column. The detection wavelength was 230 nm, and the mobile phase was composed of methanol and water (75:25, v/v).

The release of DTX from micelles was investigated by a dialysis method to ensure that the DTX-loaded nanomedicines could remain stable during the cellular experiments, and that the result of cytotoxicity could indicate the behaviors of the dosage forms (Qin et al., 2014). 0.2 mL of micellar solution was mixed with 0.8 mL of RPMI-1640 medium containing 10% FBS in a dialysis bag (molecular weight cut off =12,000–14,000 Da). The mixture was dialyzed against 20.0 mL, pH 7.4 PBS at 37 °C in a gas bath thermostatic oscillator (ZHWY-103B; ZhiCheng, Beijing, China) with gentle shaking at 100 rpm. Aliquots of 1 mL outside the dialysis bag were withdrawn at predetermined time points (1, 2, 3, 5, 7, 12, 24, and 48 h) and replaced with an equal volume of fresh PBS. The amount of released DTX was quantified by HPLC assay.

### Receptor expression study

α*7-*nAChR expression on A549 cells was determined by immunofluorescence staining. A549 cells were cultured on coverslips for 24 h, fixed with 4% (v/v) paraformaldehyde for 15 min, followed by blocked with 5% BSA for 2 h at 37 °C. Then the processed cells were incubated with primary antibody (a rat monoclonal against α7-subunit ab24644; 1:150 dilution; Abcam, Cambridge, MA) overnight at 4 °C, and further incubated with secondary antibody (fluorescein isothiocyanate (FITC)-labeled goat anti-rat IgG, 1:75 dilution; ProteinTech Group, Chicago, IL) for 1 h at 37 °C. Moreover, negative controls were treated with 1% BSA instead of monoclonal antibody to exclude the nonspecific binding. Then nucleic acid was stained with Hoechst 33258. Finally, the fluorescence of samples was analyzed with TCS SP5 confocal laser scanning microscopy (CLSM, Leica, Heidelberg, Germany).

### *In vitro* cellular uptake study by flow cytometry

A549 cells were seeded in 12-well plates (Corning, NY) at a density of approximately 5 × 10^5^ cells/well for 24 h at 37 °C. Then cells were cultured with various C6 formulations at a final C6 concentration of 100 ng/mL for 2 h at 37 °C. At the end of the treatment, cells were trypsinized, washed, re-suspended, and analyzed on FACScan flow cytometer (Becton Dickinson FACS Calibur, Mountain View, CA). The number of cells collected was 10,000, and each assay was conducted in triplicate.

To analysis the time-dependent intracellular uptake of C6-loaded micelles, cells were incubated with nanocarriers for different time periods (2, 5, 10, and 15 min), and treated as described above.

For competitive inhibition assay, 2.2 mM free ImI was pre-incubated with the cells for 1 h, followed by co-incubation with C6-loaded micelles for another 2 h. Then cells were treated according to the procedure described above.

### *In vitro* cellular uptake study by laser confocal microscopy

For confocal imaging tests, A549 cells were seeded on glass-bottom dishes for 24 h. Then cells were treated with various C6 formulations at a final concentration of 100 ng/mL for 2 h. After incubation, cells were washed, fixed with 4% paraformaldehyde. The cell nuclei were stained with Hoechst 33258 for 20 min at 37 °C. Finally, the fluorescent images of cells were captured and analyzed using CLSM.

For intracellular distribution study, after incubation with C6 formulations, cells were treated with LysoTracker Red and then fixed with 4% paraformaldehyde. Nuclear staining was performed using Hoechst 33258. Samples were observed using CLSM.

For competitive inhibition assay, cells were pretreated with 2.2 mM free ImI for 1 h prior to the addition of each formulation, and treated as described above.

### Intracellular Ca^2+^ transients analysis by laser confocal microscopy

Calcium indicator Fluo-3/AM was used to indicate the intracellular Ca^2+^ transients after the addition of nanocarriers. A549 cells were seeded on glass-bottom dishes for 24 h and incubated with Fluo-3/AM (5.0 µM) for 35 min, followed by gently washed with HEPES balanced salt solution containing calcium and magnesium (pH 7.2). Then, the fluorescence intensity was captured continuously with Leica TCS SP2 CLSM (Leica, Heidelberg, Germany) after the addition of free ImI solution (220 µM), blank PMs, or blank ImI-PMs. Blank ImI-PMs contained equivalent amount of ImI to free ImI solution. To quantitatively compare the Ca^2+^ signals of different formulations, regions of interest (ROIs) were selected randomly from the whole cell and the fluorescent images was analyzed by Leica SP2 confocal software (Leica Microsystems, Heidelberg, Germany).

### *In vivo* targeting evaluation by near-infrared fluorescence imaging

The BALB/c mice were subcutaneously inoculated with 6 × 10^6^ A549 cells in the right armpit. Then, mice randomly received 0.2 mL of free DiR, PM-DiR or ImI-PM-DiR via the tail vein (1 µg/mouse) after the tumor size was approximately 300 mm^3^ on the 45th day. For the ImI block group, mice were injected with 0.2 mL (10 mg of micelles/mL) of blank ImI-PMs at 1 h prior to the injection of ImI-PM-DiR. Animals were anesthetized with isoflurane at 1, 3, 7, 12, 24, 36, and 48 h post-injection, and the distribution of DiR-loaded nanomedicines was captured using an *in vivo* imaging system (Carestream, Fx Pro, Rochester, NY). At the end point, mice were sacrificed, followed by the collection and visualization of tumor and major organs.

### *In vitro* cytotoxicity assay by SRB

The cytotoxicity of DTX-loaded PMs against A549 cells was evaluated by SRB assay as originally described (Skehan et al., 1990). Concretely, 5000 A549 cells were seeded in each well of 96-well plates and incubated for 24 h. Then cells were exposed to various concentrations (2.5–200 nM) of DTX in different formulations. After incubation for another 48 h at 37 °C, cells were fixed with cold trichloroacetic acid, washed, and air-dried. Subsequently, the fixed cells were stained with 0.4% SRB solution for 30 min and washed with 1% acetic acid five times. Finally, the cellular bound SRB was dissolved in 10 mM Tris base solution, and the absorbance of each well at 540 nm was quantified by microplate reader (Thermo Scientific, Multiskan FC, Waltham, MA). The cell viability of cells with different treatments was calculated.

### Statistical analysis

All experiments were repeated at least three times. Quantitative data are expressed as means ± SD. Student’s *t*-test was used to evaluate significance among groups. Differences were considered significant if the value of *p* was less than .05.

## Results and discussion

### Characterization of ImI-PEG-DSPE

**Figure 1.**
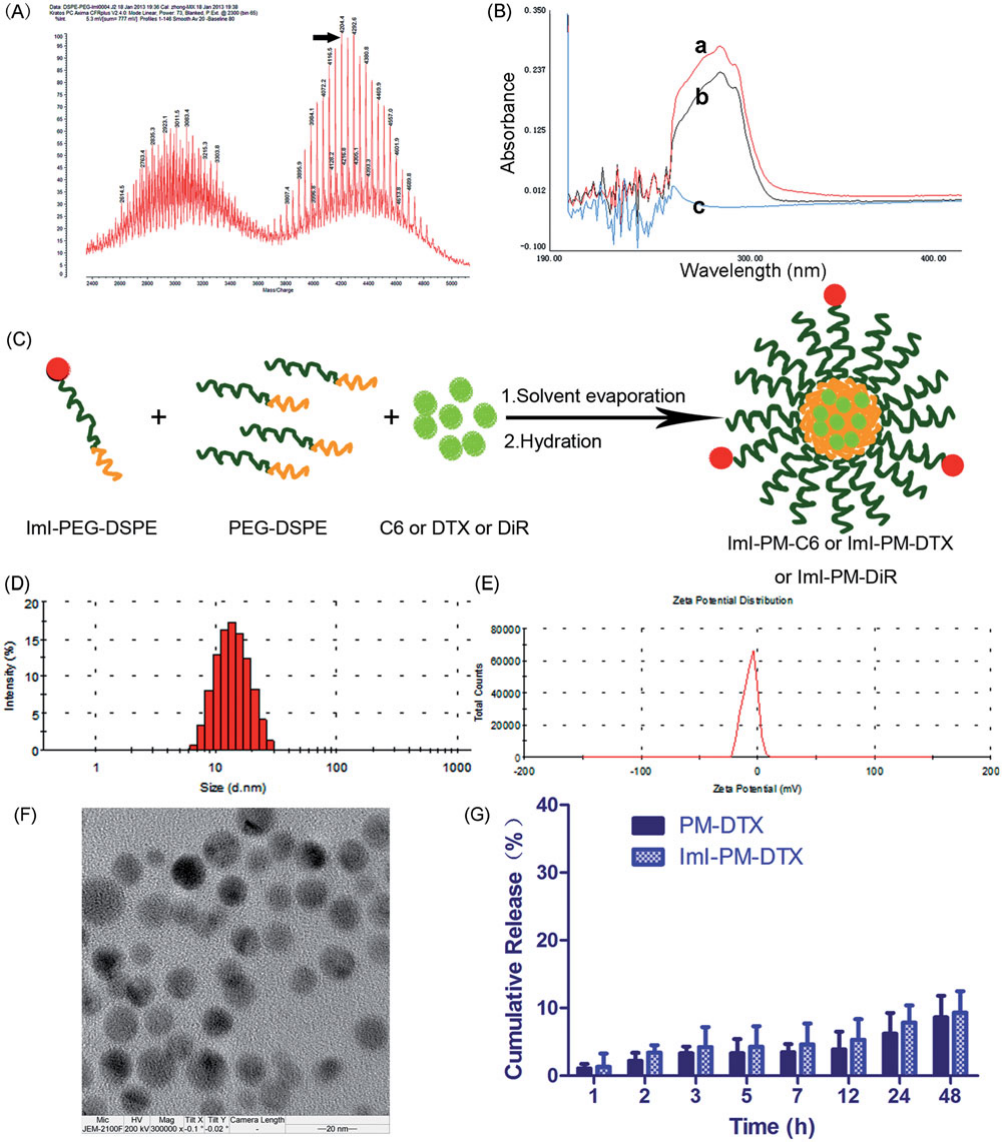
Preparation and characterization of DTX-loaded micelles. (A) MALDI-TOF mass spectra of targeting material ImI-PEG-DSPE. (B) The UV–Vis spectra scanning profiles of (a) ImI, (b) ImI-PEG-DSPE, and (c) NHS-PEG-DSPE in DMSO. (C) Schematic illustrations of preparation of drug-loaded micelles. (D) Representative size distribution profile of ImI-PM-DTX by intensity. (E) Representative zeta potential profile of ImI-PM-DTX by DLS. (F) TEM image of ImI-PM-DTX. (G) *In vitro* cumulative release of DTX from micelles in RPMI 1640 medium containing 10% FBS (*n* = 3).

The targeting material ImI-PEG-DSPE was successfully synthesized through the reaction between the NHS group of NHS-PEG-DSPE and the terminal amino group of ImI. The MALDI-TOF mass spectra of ImI-PEG-DSPE (Figure 1(A)) presented a *M*_w_ peak at 4204.4, which was in accordance with the total molecular weight of ImI (*M*_w_=1352.6) and NHS-PEG_2000_-DSPE_800_ (*M*_w_=2986.0), implying that the obtained product was the targeting copolymer ImI-PEG-DSPE (Staros et al., 1986). Moreover, the UV spectra scanning profiles of ImI, ImI-PEG-DSPE, and NHS-PEG-DSPE are shown in Figure 1(B). Compared with the UV spectrum of NHS-PEG-DSPE, ImI displayed strong absorption peaks near 284 nm, and similar absorption peaks were also observed in the UV spectrum of ImI-PEG-DSPE, indicating that ImI was successfully conjugated to the terminal NHS group of NHS-PEG-DSPE.

### Preparation and characterization of various micelles

The preparation of ImI-modified nanocarriers is schematically illustrated in Figure 1(C). As shown in Table 1 and Figure 1(D), the particle sizes of different micelle systems were similar, around 20 nm with a small polydispersity index (PDI). The nanoparticles were slightly negatively charged (about −2.00 mV, Figure 1(E)), which may be in favor of avoiding the non-specific organ uptake and accumulating in tumors efficiently (He et al., 2010). By comparison with unmodified micelles, ImI modification and its modification density exerted little effect on the physical properties of these nanocarriers, indicating that the difference in the follow-up biological experiments did not result from the different physical properties. As TEM images exhibited, ImI-PM-DTX was spherical and about 20 nm in diameter (Figure 1(F)), which was consistent with the results of DLS. Furthermore, the encapsulation efficiency of micelles for DTX, C6, or DiR was consistently greater than 95%.

**Table 1.** Characterization of various test nanomedicines (*n* = 3, mean ± SD).

Formulations		Particle size (nm)	PDI	Zeta potential (mV)	Encapsulation efficiency (%)
Blank PM		14.3 ± 1.5	0.113 ± 0.035	−2.0 ± 0.33	
Blank ImI-PM		15.4 ± 1.2	0.105 ± 0.057	−1.7 ± 0.54	
PM-C6					
0%		19.2 ± 1.3	0.111 ± 0.022	−1.8 ± 0.25	95.9 ± 3.0
ImI-PM-C6					
	0.5%	20.4 ± 1.9	0.120 ± 0.019	−1.7 ± 0.39	97.0 ± 2.5
	1%	20.2 ± 2.4	0.109 ± 0.023	−1.6 ± 0.20	96.5 ± 2.9
	5%	19.4 ± 2.6	0.135 ± 0.020	−1.6 ± 0.29	96.9 ± 3.2
	15%	21.2 ± 1.3	0.102 ± 0.041	−1.5 ± 0.21	95.7 ± 4.0
PM-DTX					
0%		17.5 ± 2.3	0.143 ± 0.051	−1.8 ± 0.33	99.1 ± 2.4
ImI-PM-DTX					
5%		19.3 ± 2.7	0.098 ± 0.038	−1.5 ± 0.18	98.0 ± 3.7

As displayed in Figure 1(G), there was no obvious difference in the release profiles of DTX from PM-DTX and ImI-PM-DTX in the medium containing 10% FBS. In this way, it was revealed that ImI modification did not alter the release character of the micelles significantly. There was only 5% of DTX released within the first 12 h, and increased to 10% in 48 h, manifesting that the DTX-loaded nanomedicines could remain stable during the cytotoxicity assay. Besides, our previous reports have indicated that both C6 and DiR have a quite slow leakage profile from nanoparticles; therefore, these fluorescent dyes could be stably loaded by micelles and are qualified for *in vitro* and *in vivo* investigation (Mei et al., 2010; Du et al., 2015).

### Receptor expression of α7-nAChR on A549 cells

**Figure 2.**
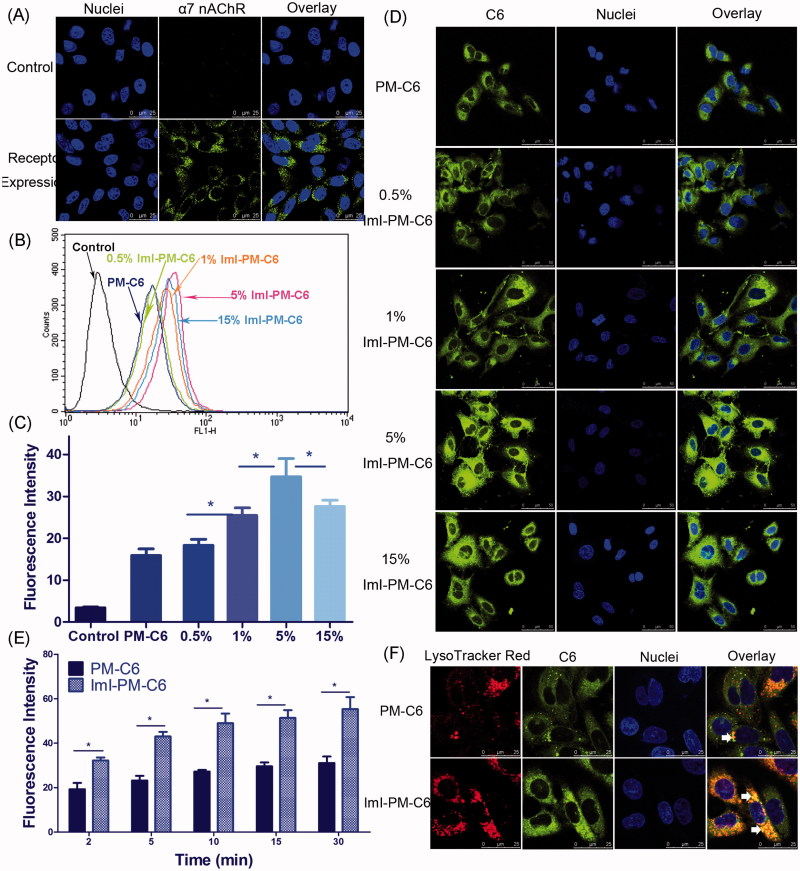
*In vitro* targeting ability of ImI-modified nanocarriers. (A) α7-nAChR expression in A549 cells observed by CLSM. Cells without incubation with primary antibody were used as negative control. Green area represents the staining of α7-nAChR. Blue region represents nuclei stained by Hoechst 33258. (B) Cellular uptake of nanocarriers with different ImI modifying densities by flow cytometry. Cells were treated with RPMI 1640 medium, PM-C6 or various ImI-PM-C6 with different molar ratios of ImI (0.5%, 1%, 5%, or 15%) for 2 h at 37 °C. (C) Quantitative results of flow cytometry analysis (*p < .05). (D) CLSM images of A549 cells incubated with PM-C6 or various ImI-PM-C6 with different molar ratios of ImI (0.5%, 1%, 5%, and 15%) for 2 h at 37 °C. Green area represents the fluorescence of C6 and blue region represents nuclei stained with Hoechst 33258. (E) Cellular uptake of PM-C6 and ImI-PM-C6 in A549 cells for different incubation time periods at 37 °C, measured by flow cytometry (*p < .05). (F) Colocalization of micelles with lysosomes. Micelles were shown in green; lysosomes were shown in red. Colocalization areas were presented as yellow spots, as representatively indicated by white arrows.

In this part, an immunofluorescence experiment was carried out to confirm α7-nAChR expression on A549 cells, since its expression was critical for the construction of α7-nAChR targeted DDS. As seen in Figure 2(A), obvious green fluorescence was observed on the cell plasma and membrane, suggesting that α7-nAChR was highly expressed on A549 cells, which was in accordance with previous research (Plummer et al., 2005; Egleton et al., 2008). Thus, A549 non-small cell lung cancer cells seem ideally suited for the DDS we designed.

### Cellular uptake of nanocarriers with different ImI modifying densities

In order to determine an optimum ImI modifying density on the micelle surface, the intracellular delivery efficiencies of targeted PEG-DSPE micelles modified by 0.5%, 1%, 5%, or 15% of ImI in molar ratio were detected by both flow cytometry and CLSM. In Figure 2(B,C), it was seen that the targeted micelles displayed higher intracellular delivery efficiency than non-modified micelles, and the micelles with 5% ImI showed the highest intracellular uptake efficiency among all nanocarriers with different modifying ratios of ImI. In other words, relatively lower ligand modifying density seemed to be more effective, which was likely attributed to the steric hindrance at the binding sites between the receptor and the ligand (Cairo et al., 2002). Moreover, our previous study has reported the similar finding for octreotide-conjugated nanomedicines (Zhang et al., 2011). Based on these results, the ImI-PM-DiR and ImI-PM-DTX modified with 5% ImI were constructed for further experiments. CLSM was utilized to visualize the cellular internalization and investigate the intracellular distribution of C6-labeled micelles directly. As we can see from Figure 2(D), the micelles modified with 5% ImI manifested the strongest internalized fluorescence. The qualitative data in confocal microscopy studies was consistent well with the quantitative one in flow cytometry experiments described above.

### Time-dependent uptake and intracellular distribution of C6-loaded nanocarriers

The kinetic uptake of PM-C6 and ImI-PM-C6 by A549 cells at 37 °C was depicted in Figure 2(E). It could be seen that the intracellular delivery efficiency exhibited a time-dependent course for both nanocarriers. Concretely, ImI-PM-C6 exhibited 1.7-, 1.9-, 1.8-, 1.7-, and 1.8-fold cellular uptake over that of PM-C6 after cultured at 37 °C for 2, 5, 10, 15, and 30 min, respectively. It was indicated that ImI modification on the surface of micelles favored the faster and more efficient internalization of this nanocarrier system into cancer cells overexpressing α7-nAChR.

Intracellular distribution study showed that ImI-PM-C6 colocalized more with lysosomes than PM-C6 after being incubated with A549 cells for 2 h (Figure 2(F)). It has been reported that cargoes would be distributed to different organelles following endocytosis, and lysosomes are the end point of the degradative pathway (Xu et al., 2013). The different lysosomal distribution profiles between non-targeting and targeting micelles suggested that the ImI modification increased the intracellular delivery of micelles to lysosomes.

### *In vitro* competition inhibition assay

**Figure 3.**
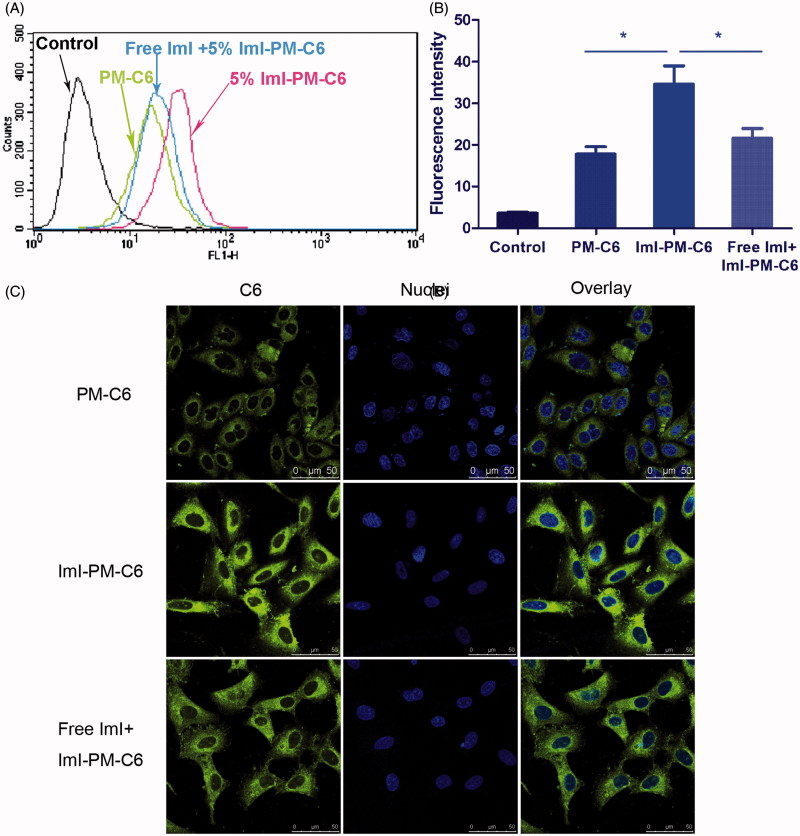
The results of competitive inhibition assay with free ImI. (A) Flow cytometry analysis of A549 cells incubated with PM-C6 or ImI-PM-C6 with 5% ImI modification ratio for 2 h at 37 °C, and the cells in the competitive group were pretreated with free ImI for 1 h. (B) Quantitative results of flow cytometry analysis (*p < .05). (C) Competition experiments conducted using confocal microscope. Green area represents the fluorescence of C6 and blue region represents nuclei stained with Hoechst 33258.

We hypothesized that the faster and more cellular uptake of ImI-PMs was probably facilitated by the existence of α7-nAChR on cell surface, hence competitive inhibition experiments were carried out to further confirm this assumption. Figure 3(A) displays the flow cytometric data of C6-loaded micelles after incubation with A549 cells for 2 h at 37 °C. It was indicated that the cellular uptake of ImI-modified micelles was much higher than that of unmodified micelles. More specifically, as can be seen from Figure 3(B), the cellular fluorescence intensity of ImI-PM-C6 was 2.19 times as high as that of passive group, and pretreatment with an excess of α7-nAChR antagonist ImI resulted in an obvious reduction of fluorescence intensity, indicating that saturation of α7-nAChR led to a decrease in internalization of ImI-PM-C6. This actually proved that the increase of cellular uptake was based on a mechanism of receptor-mediated internalization.

As shown in Figure 3(C), the ImI-PM-C6 group displayed stronger fluorescence of C6 in cytoplasm than that of the PM-C6 group, again suggesting that ImI-modified micelles had higher intracellular delivery efficiency than the non-targeted micelles. Compared with ImI-PM-C6 group, pre-incubation with free ImI caused the intracellular fluorescence intensity to decrease dramatically. These observations were generally consistent with above quantitative result obtained by flow cytometry. In conclusion, these experiments revealed that the enhanced intracellular delivery of ImI-PM-C6 was owing to the α7-nAChR-mediated internalization.

### Real-time intracellular Ca^2+^ transients

**Figure 4.**
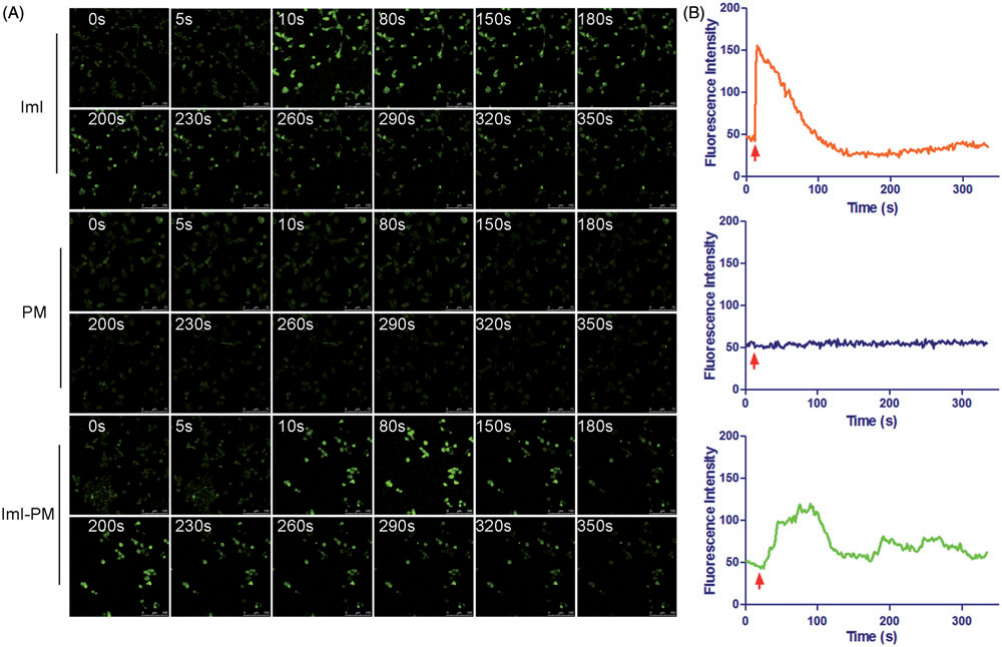
Real-time intracellular Ca^2+^ transients by fluo-3/AM fluorescence imaging technique. (A) Real-time confocal images of cytoplasmic free Ca^2+^ transients (shown as green fluorescence) in A549 cells induced by free ImI, blank PMs, or ImI-PMs. (B) Quantitative analysis of cytoplasmic free Ca^2+^ concentrations. Each point on the plots represents mean fluorescence intensity obtained from at least 10 randomly selected ROIs. Red arrows indicate the time for addition of free ImI, blank PMs, or ImI-PMs.

The whole cellular uptake of nanocarriers is generally consisted of two processes, i.e. surface binding on cell membrane and the following internalization. Then, to investigate the effect of ImI modification on the interaction of micelles with A549 cells, an intracellular Ca^2+^ transients study was conducted to monitor the molecular interaction in real time by fluo-3/AM fluorescence imaging technique (Ween et al., 2010). Extensive studies have showed that α7-nAChR is a ligand-gated calcium channel with high permeability for Ca^2+^. Ligand binding (such as α-conotoxions) induces a conformational change of the receptor, affording a flow of Ca^2+^ with downstream signaling cascades (Zia et al., 2000; Arredondo et al., 2002). Therefore, the molecular interaction between ImI-PMs and α7-nAChR can be monitored by the effects of α-conotoxion ImI on cytoplasmic free Ca^2+^ concentration ([Ca^2+^]i), which could be indicated by the fluorescence intensity of the calcium indicator Fluo-3/AM. As exhibited in Figure 4, blank unmodified PMs, blank ImI-PMs, and free ImI solution were added to A549 cells with high α7-nAChR expression, and the fluorescence signals that reflected the interaction between micelles and cells were recorded. In the positive control group, free ImI triggered the instant increase and subsequent gradual decline in [Ca^2+^]i. This instant increase of cytoplasmic [Ca^2+^]i following the addition of ImI is likely due to the quick calcium release from cellular calcium stores such as mitochondria and endoplasmic reticulum which play important and interactive roles in the regulation of Ca^2+^ homeostasis. Afterwards, continuous α7-nAChR blockade by ImI induced a progressive decline in [Ca^2+^]i level. Similar to free ImI, the targeted micelles modified with equivalent ImI caused slow but obvious increase and subsequent decrease in [Ca^2+^]i. It could be speculated that free ImI swiftly diffused to the surface of cells and instantly bound with α7-nAChR, whereas the ImI-modified micelles diffused more slowly due to its larger size, resulting in a slower effect on [Ca^2+^]i. Additionally, a slight fluctuation in [Ca^2+^]i level could be seen in A549 cells treated with ImI-PMs. A possible reason may be the steric hindrance at the binding sites between the receptor and ligand, leading to a temporarily unstable bond. However, the Ca^2+^ signals started to level off from 300 s on, indicating that a firm binding was formed between α7-nAChR and ImI-PMs. By contrast, blank unmodified PMs did not cause obvious [Ca^2+^]i change as compared with other treatments.

Therefore, it demonstrated that ImI-PMs could enhance the interaction between nanocarriers and tumor cells highly expressing α7-nAChR. This interaction was considered as the key factor that rendered faster and more efficient internalization of ImI-PMs into cytoplasm. Moreover, it is worth noting that it is necessary to choose an appropriate ligand modification density on the micelle surface, which could minimize the adverse impact on the cellular uptake caused by steric hindrance.

### *In vivo* targeting evaluation

**Figure 5.**
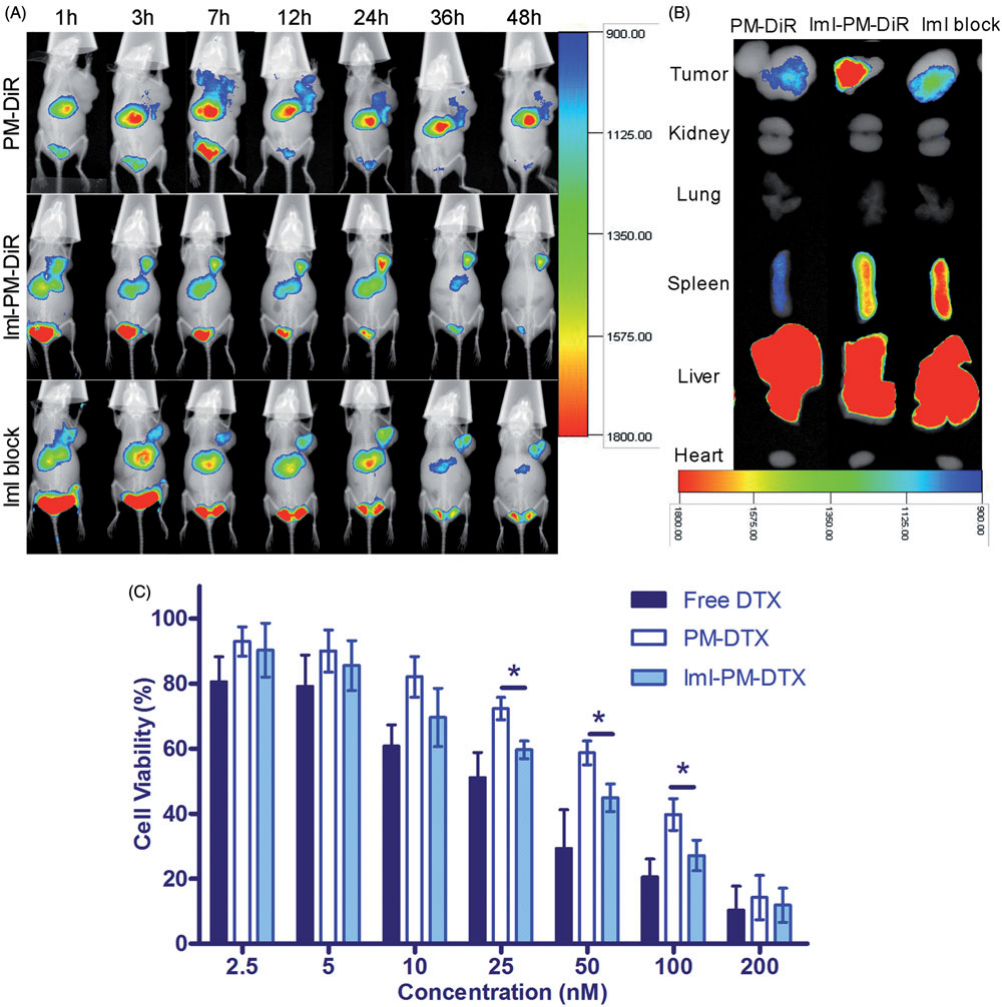
(A) *In vivo* distribution of ImI-modified micelles in A549 tumor-bearing mice. Mice were intravenously administrated with 0.2 mL (5 μg DiR/mL) of PM-DiR, ImI-PM-DiR, ImI-PMs plus ImI-PM-DiR (defined as the ImI block group). (B) *Ex vivo* fluorescent images of tumors and major organs excised at the end point. (C) *In vitro* cytotoxicity of free DTX, PM-DTX, and ImI-PM-DTX at different concentration against A549 cells for 48 h (*p < .05).

To evaluate the targeting effect of ImI-modified micelles and its tissue distribution *in vivo*, DiR, a near-infrared fluorescent indicator, was encapsulated into ImI-modified micelles and unmodified micelles. As observed in Figure 5(A), the fluorescence signals of PM-DiR and ImI-PM-DiR at the tumor sites were both increased with time during the first 12 h, while ImI-PM-DiR distributed faster and more at tumor site than PM-DiR at each tested time point. The PM-DiR passively accumulated in the solid tumors because of the enhanced permeation and retention (EPR) effects (Maeda, 2010). Meanwhile, ImI modification on the surface of micelles further increased the drug accumulation into tumor sites through the special interaction between ImI and its receptor α7-nAChR on the basis of EPR effect. Furthermore, to verify the foregoing inference, α7-nAChR was saturated by blank ImI-PMs for 1 h before the injection of ImI-PM-DiR. Consistent with results obtained *in vitro* (Figure 3), the tumoral accumulation of micelles in block group was significantly decreased at all tested time points when compared with ImI-PM-DiR group. In the *ex vivo* images (Figure 5(B)), a remarkably higher accumulation of micelles was observed in the tumor of ImI-PM-DiR group than that of other groups. Moreover, all DiR formulations preferentially distributed in reticuloendothelial system (RES) organs, including liver and spleen, which was probably caused by the clearance of the RES (Storm et al., 1995). In this way, it was proved *in vivo* that the modification of ImI could facilitate the tumoral delivery of nanocarriers for tumors overexpressing α7-nAChR via a receptor-mediated uptake.

Additionally, based on the *in vitro* and *in vivo* results, it could be deduced that this receptor would be partially saturated, thus leading to a decrease in the uptake of targeted micelles. This receptor saturation phenomenon is similar to the previous findings for the transferrin receptor targeted nanoparticles (Du et al., 2015). It is worth considering for more efficient targeted drug delivery system.

### *In vitro* tumor cell inhibition of ImI-PM-DTX

Figure 5(C) illustrates the viability of A549 cells after being incubated with different DTX formulations for 48 h. A dose-dependent effect was found for both free DTX and micelle formulations. Free DTX (IC50 = 29.3 ± 5.06 nM) exhibited the strongest cytotoxicity among all the treatments. One possible reason is that the DTX molecules diffused directly into cells, whereas the endocytosis of micelles was slower than the cellular uptake of free DTX, resulting in a lower effect. It was clear that ImI-PM-DTX (IC50 = 39.1 ± 5.97 nM) showed a significantly better inhibitive effect on cell proliferation compared with PM-DTX (IC50 = 70.0 ± 6.86 nM) at a concentration range of 25–100 nM (*p* < .05). Moreover, the cytotoxicity of blank polymer micelles (PMs and ImI-PMs) against A549 cells were also evaluated (data not shown). As a result, both blank micelles hardly displayed inhibitive effect at all tested concentrations, revealing the biocompatibility of the polymeric micelles. In sum, this finding revealed that the modification of micelles with ImI could increase the intracellular delivery of DTX, leading to higher activities in the inhibition of tumor proliferation, which was in accordance with the enhanced cellular uptake in flow cytometry and confocal microscopy analysis discussed above.

In our research, an ImI-modified nanocarrier was constructed to target α7-nAChR overexpressed NSCLC. Extensive studies have reported that the expression of α7-nAChR exhibited a significant upregulation in NSCLC adenocarcinoma, squamous cell carcinoma, as well as large cell carcinoma when compared with normal tissues. Moreover, recent data suggested that α7-nAChR is significantly more expressed in squamous carcinoma than in adenocarcinoma. Among this histological subtype, smokers showed the highest upregulation (Paleari et al., 2009). As a consequence of α7-nAChR expression specificity and significance, based on our results, α7-nAChR targeted nanocarrier may be a potential delivery system for NSCLC, especially for squamous in smokers. It is worth mentioning that, in view of individual difference in clinical practice, the expression level of α7-nAChR should be confirmed by human biopsies taken from patients with lung cancer before doctors make a therapeutic decision.

## Conclusions

In this study, a novel DTX-loaded and α-conotoxin ImI-modified micelle system was constructed and characterized. The *in vitro* and *in vivo* data indicated that ImI-modified PEG-DSPE micelles exhibited higher intracellular drug delivery over non-targeted micelles on α7-nAChR overexpressed A549 non-small cell lung cancer cells via the receptor-mediated internalization. Moreover, ImI-PM-DTX achieved greater inhibition of tumor proliferation *in vitro* than that of unmodified micelles, due to the facilitation of the receptor-specific intracellular transport of DTX into A549 cells. Based on the above observations, it could be concluded that ImI-modified micelles may be a potential targeted drug delivery system for α7-nAChR overexpressed NSCLC by enhancing the intracellular uptake of hydrophobic anticancer drugs.
